# Large-Scale Analysis of the Diversity and Complexity of the Adult Spinal Cord Neurotransmitter Typology

**DOI:** 10.1016/j.isci.2019.09.010

**Published:** 2019-09-10

**Authors:** Andrea Pedroni, Konstantinos Ampatzis

**Affiliations:** 1 Karolinska Institutet, Department of Neuroscience, 171 77 Stockholm, Sweden

**Keywords:** Biological Sciences, Neuroscience, Systems Neuroscience, Neuroanatomy

## Abstract

The development of nervous system atlases is a fundamental pursuit in neuroscience, since they constitute a fundamental tool to improve our understanding of the nervous system and behavior. As such, neurotransmitter maps are valuable resources to decipher the nervous system organization and functionality. We present here the first comprehensive quantitative map of neurons found in the adult zebrafish spinal cord. Our study overlays detailed information regarding the anatomical positions, sizes, neurotransmitter phenotypes, and the projection patterns of the spinal neurons. We also show that neurotransmitter co-expression is much more extensive than previously assumed, suggesting that spinal networks are more complex than first recognized. As a first direct application, we investigated the neurotransmitter diversity in the putative glutamatergic spinal V2a-interneuron assembly. These studies shed new light on the diverse and complex functions of this important interneuron class in the neuronal interplay governing the precise operation of the central pattern generators.

## Highlights

• The structural organization of the adult zebrafish spinal cord is highly diverse
• Equal number of ascending and descending neurons are excitatory or inhibitory
• Several (∼15%) spinal neurons express multiple neurotransmitter phenotypes
• Part of the glutamatergic V2a-INs co-express GABA, glycine, or acetylcholine

## Graphical Abstract

**Figure:**
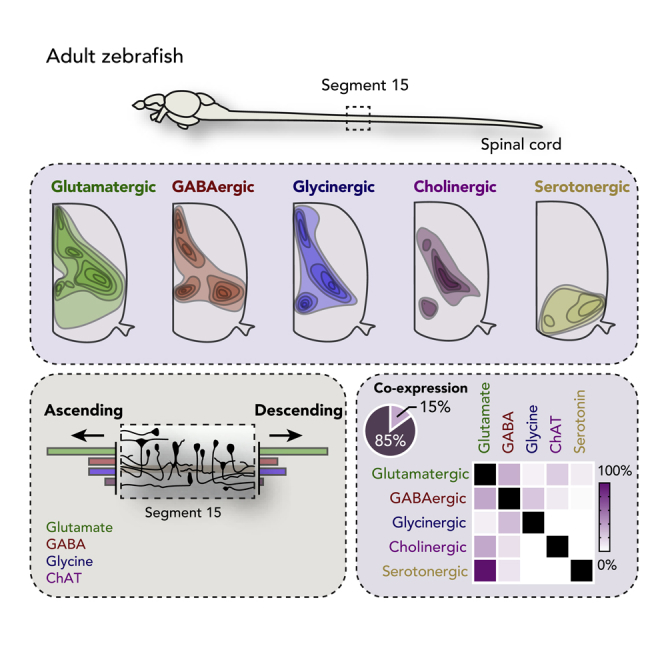

## Introduction

Neuronal networks in the spinal cord are able and sufficient to generate and control movements and receive and process sensory information (Arber, 2012, Goulding, 2009, Grillner and Jessell, 2009, Kiehn, 2016). Their functionality depends on the correct specification of different classes of neurons during development (Alaynick et al., 2011, Arber, 2012, Goulding, 2009, Jessell, 2000), which allows them to establish precise connections. Spinal neurons derive from specific progenitor pools in the spinal cord and express precisely a combination of transcription factors (Alaynick et al., 2011, Arber, 2012, Goulding, 2009, Jessell, 2000). Their developmental diversification is well understood (Arber, 2012, Goulding, 2009, Jessell, 2000, Kiehn, 2016), but it is not clear how several functional characteristics of these cells are specified. A particularly important determinant of a neuron's functionality is its neurotransmitter phenotype.

Neuronal communication involves the release and uptake of specific neurotransmitters (Rogawski and Barker, 1986, Schwartz, 2000), endogenous chemical messengers used in intercellular signaling across synapses. The vertebrate nervous system uses neurotransmitters including glutamate, γ-aminobutyric acid (GABA), glycine, and acetylcholine to mediate biological functions such as sensory perception and to generate complex behaviors (Rogawski and Barker, 1986, Schwartz, 2000, Unwin, 1993). Neurons can be classified as excitatory, inhibitory, or modulatory based on their neurotransmitter phenotypes. Therefore, the adoption of a specific neurotransmitter system by a given neuron type defines its identity. To understand specific neurons' roles in integrated neural networks, one must identify the transmitters they use to modulate their targets. Neuroanatomically precise maps of neurotransmitter typology distributions facilitate this by revealing correlations between the anatomical and functional neuronal architectures.

The zebrafish is an important model organism for high-throughput studies on neuronal circuits' functions and behavior, and much is known about the different cell types in the zebrafish spinal cord (Ampatzis et al., 2013, Bernhardt et al., 1990, Bernhardt et al., 1992, Björnfors and El Manira, 2016, Bradley et al., 2010, Böhm et al., 2016, Djenoune et al., 2017, Hale et al., 2001, Higashijima et al., 2004a, Higashijima et al., 2004b, Kimura et al., 2008, Liao and Fetcho, 2008, McLean et al., 2007, Menelaou et al., 2014, Satou et al., 2012, Stil and Drapeau, 2016). However, the number and identity of the spinal excitatory and inhibitory neurons that process sensory-related information are unknown, as are the neurotransmitter identities of the neurons that control and gate motor commands. This is a critical limitation because neuronal activity depends strongly on neurotransmitter identity. To overcome this limitation, we conducted the first systematic quantitative neurotransmitter phenotype analysis of neurons in adult zebrafish spinal networks by using an anatomical high-throughput strategy to investigate individual populations of spinal excitatory, inhibitory, and modulatory neurons. Our results reveal a previously unsuspected co-expression of different neurotransmitters in spinal cord neurons, and we show that these multi-phenotype neurons are far more numerous and widely distributed in the spinal cord than previously assumed. We use this comprehensive neurotransmitter map to describe the co-existence of classical neurotransmitters in the presumed putative glutamatergic V2a interneuron population, revealing an unsuspected neurotransmitter co-expression within this cohered group of interneurons. The comprehensive neurotransmitter typology atlas presented here reveals an unforeseen diversity, complexity, and dynamics in the principles that govern the structural organization of the adult zebrafish spinal cord and provides an anatomical framework to guide further functional dissection of spinal neuronal circuits.

## Results

### Neuronal Composition of the Adult Spinal Cord

**Figure 1:**
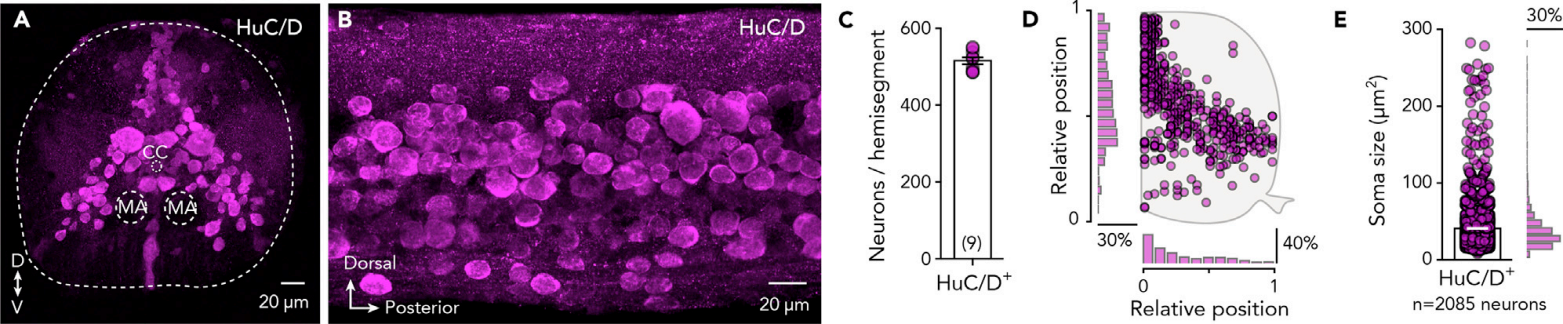
Neuroanatomy of Adult Zebrafish Spinal Cord (A and B) Transverse section and whole-mount adult zebrafish spinal cord showing the expression of the pan-neuronal marker HuC/D^+^ neurons. (C) Quantification of the number of spinal neurons (HuC/D^+^) located in adult spinal cord hemisegment (segment 15). (D) Spatial distribution of the HuC/D^+^ neurons with the medio-lateral and dorsoventral density plot from one adult zebrafish spinal hemisegment (*n* = 478 labeled cells). (E) Quantification and distribution of the HuC/D^+^ neurons soma size (*n* = 2,085 neurons). Data are presented as mean ± SEM. CC, central canal; MA, Mauthner axon. For antibodies information, see also Table S1.

We first sought to determine the number of neurons in a representative hemisegment (segment 15) of the adult zebrafish spinal cord by using immunohistochemistry to detect the expression of the pan-neuronal marker HuC/D. This revealed that neurons were distributed throughout the adult spinal cord, from the most dorsal and medial part to the most lateral aspects (Figures 1A, 1B, and 1D). However, only a small fraction of the labeled neurons was observed in the ventral part of the spinal hemisegment and the dorsal neuropil area (Figure 1D). Detailed quantification showed that an adult zebrafish spinal hemisegment contains 515.7 ± 8.865 neurons (segment 15; Figure 1C). Although the soma sizes of the labeled spinal neurons varied considerably, the vast majority were small or medium sized (41.17 ± 0.63 μm^2^, *n* = 2085 neurons; Figure 1E). These results show that the adult spinal cord has a well-defined and diverse neuron population and provides a starting point for further characterizing the neurochemical architecture of adult zebrafish spinal cord networks.

### Neurotransmitter Typology of Spinal Cord Neurons

**Figure 2:**
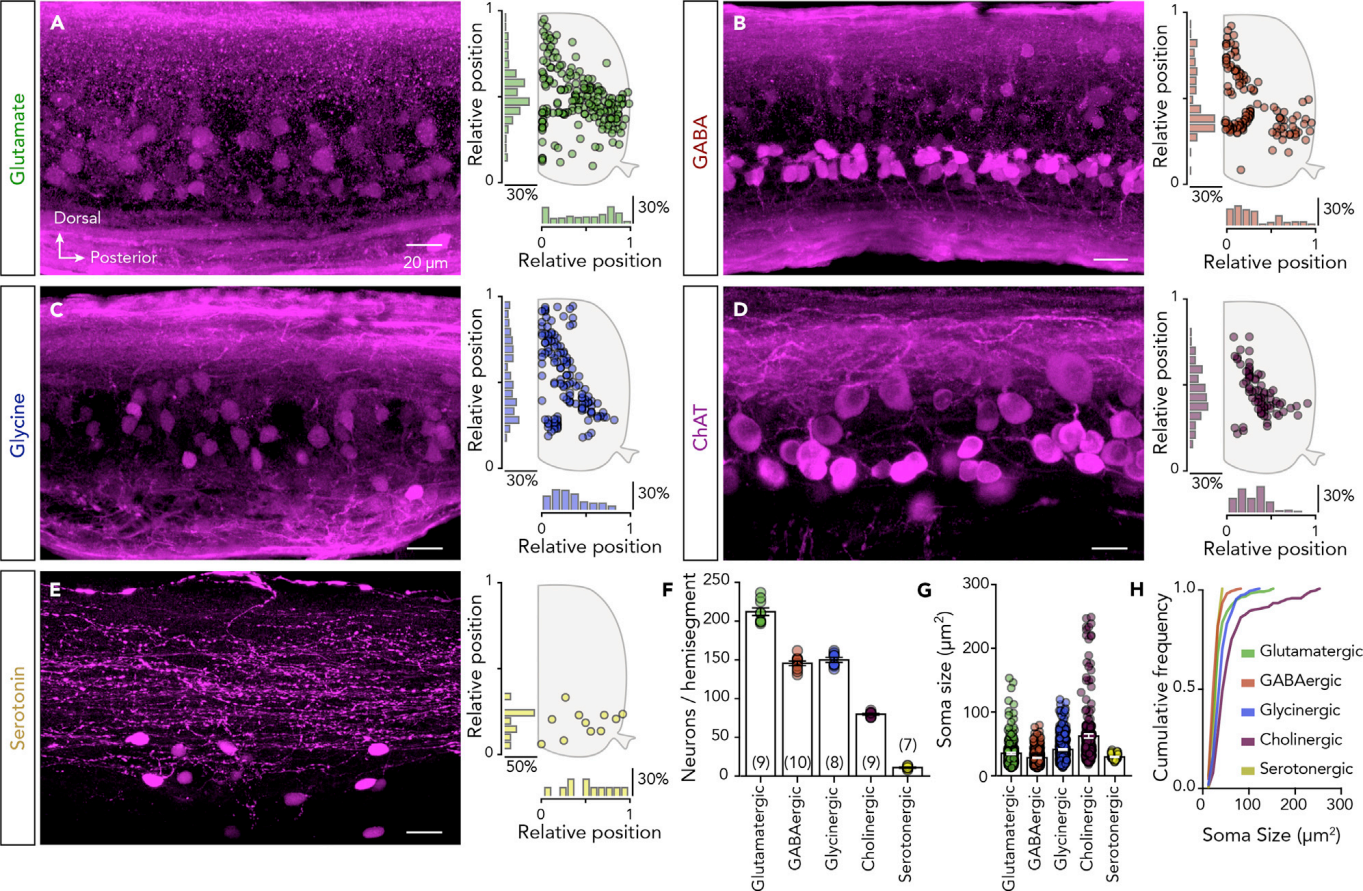
Neurotransmitter Phenotypes of the Adult Zebrafish Spinal Neurons (A–E) Representative whole-mount photomicrographs showing part of the immunolabeled cells for glutamate, GABA, glycine, ChAT, and serotonin, followed by a schematic representation of the spatial distribution with the corresponding medio-lateral and dorsoventral density plots from a single adult zebrafish spinal cord hemisegment. (F) Quantification of the total number of the labeled neurons expressing a specific neurotransmitter phenotype. (G and H) Quantification and cumulative frequency of labeled neurons soma size. Data are presented as mean ± SEM. For related data and antibodies information, see also Figures S1 and S5 and Table S1.

Despite previous studies on zebrafish spinal neurotransmitter phenotypes (Higashijima et al., 2004a, Higashijima et al., 2004b), the number, size, and location of the neurons involved in the spinal networks are currently unknown. Therefore, to provide a reliable foundation for computational modeling and to identify new targets for electrophysiological recordings, we attempted to create a complete and detailed map of the neurotransmitter typology in the adult zebrafish spinal cord. All spinal neurons were found to express one of the classical neurotransmitters considered in this work (glutamate, GABA, glycine, acetylcholine, and serotonin; Figures 2A–2D). In keeping with previous reports, we detected no dopaminergic or noradrenergic spinal neurons (McLean and Fetcho, 2004; Figure S1). The glutamatergic, GABAergic, and glycinergic neurons had similar distributions (Figures 2A–2C), whereas cholinergic neurons were almost absent from the dorsal part of the spinal cord (Figure 2D) and serotonergic neurons were observed only in the ventral part (Figure 2E). Quantification of individual neuronal classes revealed that most neurons are glutamatergic (212.1 ± 5.01 neurons, *n* = 9 zebrafish), GABAergic (145.5 ± 2.918 neurons, *n* = 10 zebrafish), and glycinergic (150 ± 3.179 neurons, *n* = 8 zebrafish; Figure 2F). Cholinergic neurons constitute a smaller population (79.78 ± 1.024 neurons, *n* = 9 zebrafish), and only few serotonergic neurons were found (11 ± 0.755 neurons, *n* = 7 zebrafish; Figure 2F). Finally, soma size measurements showed that all these neuronal populations had similar mean soma sizes; however, the cholinergic and serotonergic neurons displayed the greatest and least soma size variability, respectively (glutamatergic: 35.11 ± 1.57 μm^2^, *n* = 206 neurons; GABAergic: 27.99 ± 0.525 μm^2^, *n* = 407 neurons; glycinergic: 41.04 ± 0.953 μm^2^, *n* = 379 neurons; cholinergic: 62.54 ± 3.427 μm^2^, *n* = 208 neurons; serotonergic: 29.5 ± 0.815 μm^2^, *n* = 37 neurons; Figures 2G and 2H). The distributions of the different neurotransmitter-expressing neurons in the adult zebrafish spinal cord are thus highly stereotypic and heterogeneous.

### Neurotransmitter Phenotypes of Projecting Spinal Neurons

**Figure 3:**
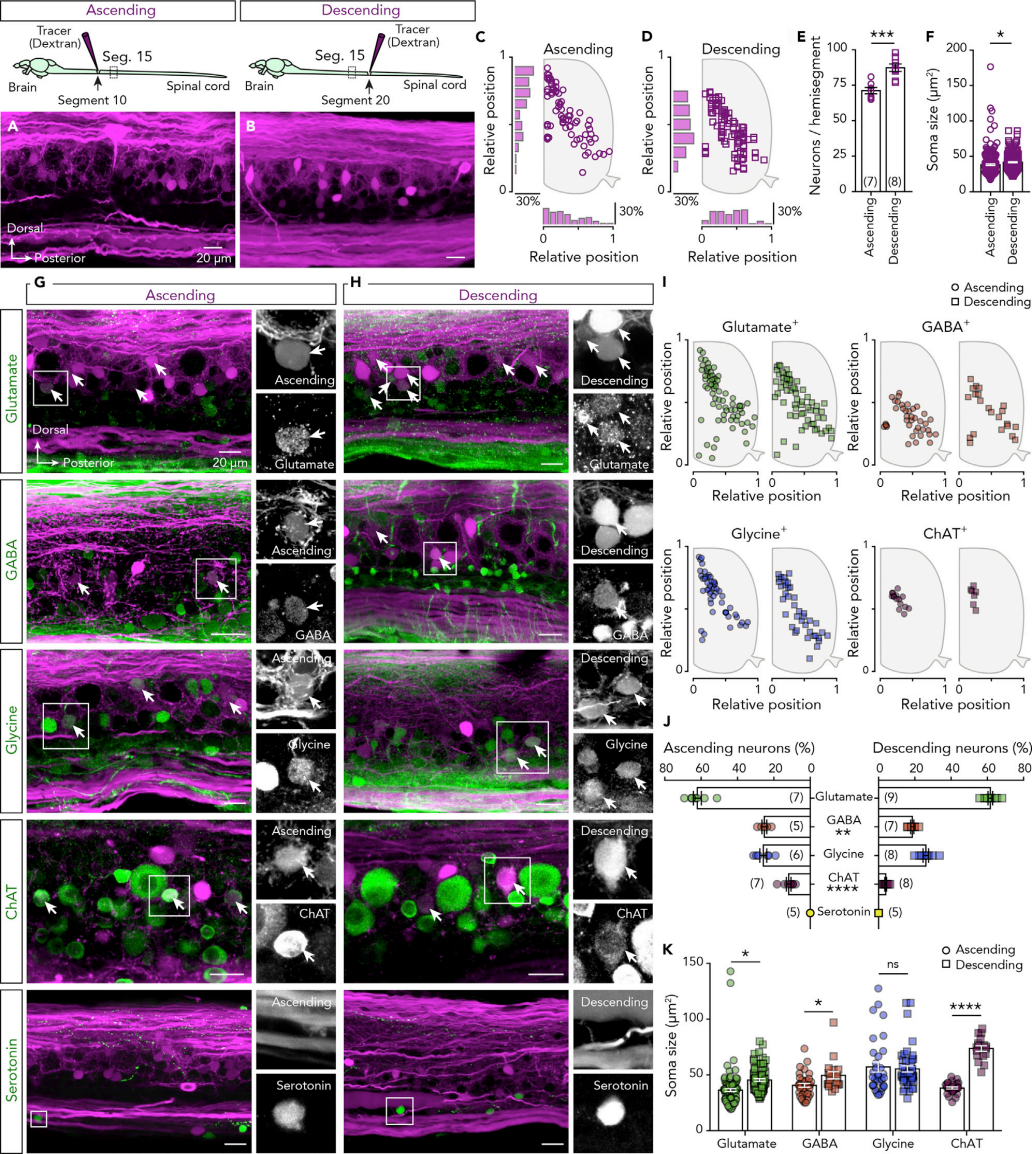
Neurotransmitter Phenotype of Projecting Neurons (A and B) Injection of a dextran tracer in segment 10 or 20 reveals the ascending and descending spinal projecting neurons, respectively, located in spinal cord segment 15. (C and D) Setting positions of the tracer-labeled ascending (circles) and descending (squares) neurons that project to the rostral or caudal part of the spinal cord revealed in one representative preparation. (E) Quantification of the total number of ascending and descending neurons detected in the spinal cord hemisegment. (F) Plot showing the soma sizes of the tracer-labeled ascending and descending neurons (Ascending: *n* = 217 neurons; Descending: *n* = 185 neurons). (G and H) Double staining between ascending or descending traced neurons (magenta) with glutamate, GABA, glycine, ChAT, and serotonin (green). Arrows indicate the double-labeled neurons. On the right side, there are single channel magnifications of the boxed area. (I) Spatial distribution of the ascending (circles) and descending (squares) traced neurons that express a specific neurotransmitter phenotype. (J) Quantification of percentage of tracer-positive ascending and descending projecting neurons expressing each neurotransmitter phenotype. (K) Soma sizes of the tracer-positive ascending (circles) and descending (squares) projecting neurons. Data are presented as mean ± SEM; *P < 0.05; **P < 0.01; ***P < 0.001; ****P < 0.0001; ns, non-significant. For antibodies information, see also Table S1.

The projection patterns of the spinal cord neurons must be understood to explain their inputs to the circuits that process sensory information and control motor behaviors. We therefore used an anatomical tracing technique to determine the positions, number, and sizes of the projecting spinal neurons. Specifically, we identified every neuron located in hemisegment 15 that projects over five or more spinal segments to a rostral (ascending) or caudal (descending) spinal cord (Figures 3A and 3B). We found that most ascending neurons (∼75%) are located in the dorsal and medial part of the spinal cord, whereas the descending neurons are located in the motor column area (Figures 3C and 3D). Furthermore, the ascending neurons comprise a significantly smaller population (71.29 ± 2.212 neurons) than the descending neurons (87.38 ± 2.639 neurons; unpaired t test: t = 4.594, df = 13, P = 0.0005; Figure 3E), and their soma sizes differ (ascending: 38.33 ± 1.106 μm^2^, *n* = 217 neurons; descending: 41.76 ± 0.894 μm^2^, *n* = 185 neurons; unpaired t test: t = 2.354, df = 400, P = 0.0191; Figure 3F).

To determine the projecting neurons' neurotransmitter phenotypes, we combined the tracing with immunolabeling of the classical neurotransmitters (Figures 3G and 3H). This revealed that the ascending neurons located in the most dorsal area of the spinal cord are glutamatergic and glycinergic (Figure 3I), whereas GABAergic and cholinergic projecting neurons are co-distributed in the medial and ventral parts of the spinal cord (Figure 3I). In addition, no serotonergic neurons displayed ascending or descending projections extending over more than five segments (Figures 3G and 3H). Quantitative transmitter phenotype analyses showed that most projecting neurons are glutamatergic (ascending: 62.12 ± 2.2%, n = 7 zebrafish; descending: 61.67 ± 1.3%, *n* = 9 zebrafish; Figure 3J), whereas GABAergic (ascending: 25.29 ± 1.323%, *n* = 5 zebrafish; descending: 18.6 ± 0.858%, *n* = 7 zebrafish) and glycinergic (ascending: 25.87 ± 1.899%, *n* = 6 zebrafish; descending: 26.09 ± 1.542%, *n* = 8 zebrafish) neurons form notably smaller populations. We also found a few projecting cholinergic neurons (ascending: 11.87 ± 1.297%, *n* = 7 zebrafish; descending: 3.96 ± 0.506%, *n* = 8 zebrafish; Figure 3J). With GABAergic (unpaired t test: t = 4.45, df = 10, P = 0.0012; Figure 3J) and cholinergic (unpaired t test: t = 5.975, df = 13, P < 0.0001; Figure 3J) projecting neurons to exhibit significant differences. Our analysis suggests that similar patterns of excitation and inhibition are delivered to the rostral and caudal parts of the spinal cord. Finally, to determine whether different neuron types innervate the rostral and caudal parts of the spinal cord, we quantified the soma sizes of projecting neurons with respect to their neurotransmitter phenotypes (Figure 3K). Although in most cases the soma sizes of the ascending and descending neurons were significantly different (unpaired t test: glutamatergic: t = 2.33, df = 143, P = 0.021; GABAergic: t = 2.652, df = 52, P = 0.01; Figure 3K), only the cholinergic neurons displayed populations with non-overlapping sizes (unpaired t test: t = 15.22, df = 43, P < 0.0001; Figure 3K), suggesting that they constitute two distinct projecting subpopulations.

### Spinal Neurons Express Multiple Neurotransmitter Phenotypes

**Figure 4:**
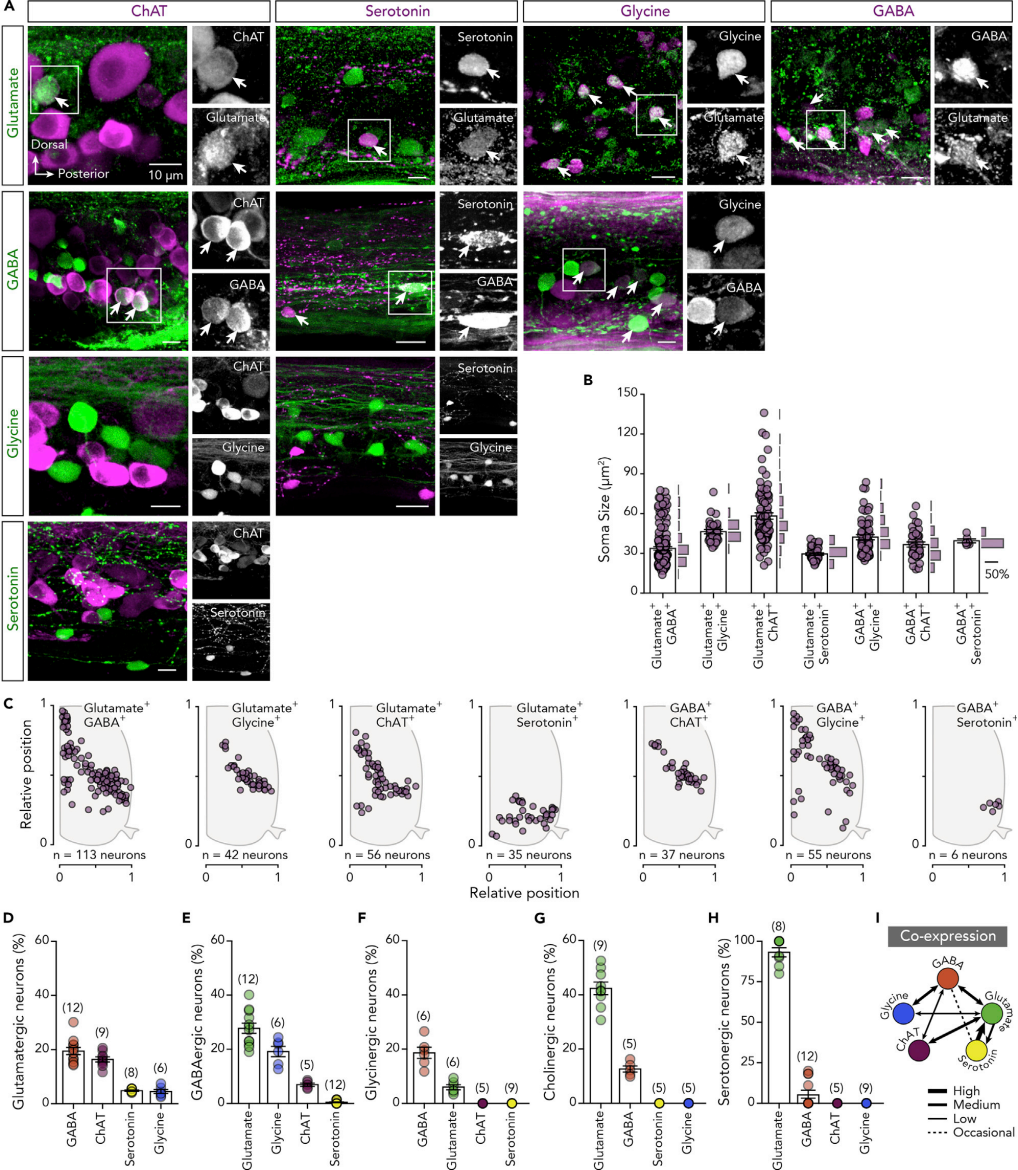
Spinal Cord Neurons Express Multiple Neurotransmitter Phenotypes (A) Whole-mount double immunolabeling between glutamate, GABA, glycine, ChAT, and serotonin. In black and white are single channel images of the merged images. Arrows indicate the double-labeled neurons. (B and C) Soma sizes and spatial distribution of the detected double-stained neurons in the adult zebrafish spinal cord hemisegment. (D–H) Quantification of percentage of glutamatergic, GABAergic, glycinergic, cholinergic (ChAT^+^), and serotonergic neurons that co-express other neurotransmitters. (I) Schematic relationship of the neurotransmitters co-expression from the adult zebrafish spinal cord neurons. Data are presented as mean ± SEM. For related data and antibodies information, see also Figures S2 and S3 and Table S1.

Our analysis of neurotransmitter phenotypes in adult zebrafish spinal neurons suggested that the total number of neurons expressing a specific classical neurotransmitter is ∼600. Since we detected 515 neurons in each spinal cord hemisegment, this possibly implies that some spinal cord neurons (∼15%) express multiple neurotransmitter phenotypes. To test this hypothesis, the extent of co-expression was determined using binary neurotransmitter immunodetection. We found that several neurons co-express two neurotransmitter phenotypes (Figure 4A) and that these neurons have specific distribution patterns in the spinal cord (Figure 4C). However, we found no co-expression of ChAT with serotonin and glycine or of glycine with serotonin (Figure 4A). To determine whether neurons with dual neurotransmitter phenotypes comprise separate neuronal subpopulations that settle at distinct positions in the spinal cord, we measured the somas of double-labeled neurons (Figure 4B).

Next, we determined the extent of dual neurotransmitter expression in different neuron populations. We found that a notable proportion of glutamatergic neurons are GABAergic (19.46 ± 1.302%, *n* = 12 zebrafish), cholinergic (16.37 ± 0.927%, *n* = 9 zebrafish), serotonergic (4.814 ± 0.224%, *n* = 8 zebrafish), or glycinergic (4.507 ± 0.758%, *n* = 6 zebrafish; Figure 4D). In addition, many GABAergic neurons co-express glutamate (27.76 ± 1.879%, *n* = 12 zebrafish) or glycine (19.2 ± 1.922%, *n* = 6 zebrafish), and a few were immunolabeled for choline acetyltransferase (ChAT; 6.965 ± 0.602%, *n* = 5 zebrafish) or serotonin (0.413 ± 0.185%, *n* = 12 zebrafish; Figure 4E). However, glycinergic neurons were observed to co-express only GABA (18.67 ± 2.057%, *n* = 6 zebrafish) and glutamate (6.008 ± 0.9372%, *n* = 6 zebrafish; Figure 4F), as do cholinergic neurons (GABA: 12.62 ± 1.15%, *n* = 5 zebrafish; Glutamate: 42.42 ± 2.311%, *n* = 9 zebrafish; Figure 4G). Finally, many serotonergic neurons were found glutamatergic (93.19 ± 2.83%, *n* = 8 zebrafish), and a few occasionally (4 of 12 zebrafish) to co-express GABA (5.623 ± 2.47%, *n* = 12 zebrafish; Figure 4H). Most notably, the GABAergic/serotonergic neurons were found to consist of a subpopulation of the serotonergic neurons that possess larger soma sizes (Figure 4B) and their distribution is restricted to the ventral and lateral part of the spinal cord (Figure 4C).

The validity of our observations was confirmed also by immunohistochemistry using transgenic animal lines (*VGlut2a:GFP; GAD1b:GFP; GlyT2:GFP* and *Tph2:GFP*) to detect the proposed neurotransmitter phenotypes (see Transparent Methods, Figures S2 and S5). To verify that spinal neurons can co-release different neurotransmitters, we performed *in situ* hybridization experiments using the sensitive RNAscope method to detect individual mRNAs for the vesicular glutamate transporter (VGlut2a, *slc17a6b*) found in neurons that release glutamate as a transmitter (Shigeri et al., 2004), the vesicular acetylcholine transporter (vAChT, *slc18a3b*; a specific transporter of cholinergic neurons, Weihe et al., 1996), and the vesicular GABA transporter (vGAT, *slc32a1*; also known as vIAAT, vesicular inhibitory amino acid transporter) a transporter for both GABAergic and glycinergic neurons (Chaudhry et al., 1998, Wojcik et al., 2006, Figures S3A–S3C). We observed the presence of different combinations (co-localizations) of the vesicular transporter mRNA puncta in individual neurons (Figures S3D–S3G), confirming that adult spinal cord neurons host the cellular machinery needed to store and release (co-transmit) different classical neurotransmitters. Interestingly, we also observed small populations of spinal neurons containing all three vesicular transporter mRNA puncta (Figure S3G), suggesting the existence of triple co-transmission. We verified this observation by immunohistochemistry and investigated the distribution and soma sizes of spinal cholinergic neurons that co-express GABA and glutamate (Figure S3H).

Together, these data provide the first evidence that the characterization of neurons as being either excitatory or inhibitory is an oversimplification that does not properly reflect the neurotransmitter complexity of neuronal populations in the vertebrate spinal cord (Figure 4I).

### V2a Interneuron Neurotransmitter Diversity: A Proof-of-Concept Analysis

**Figure 5:**
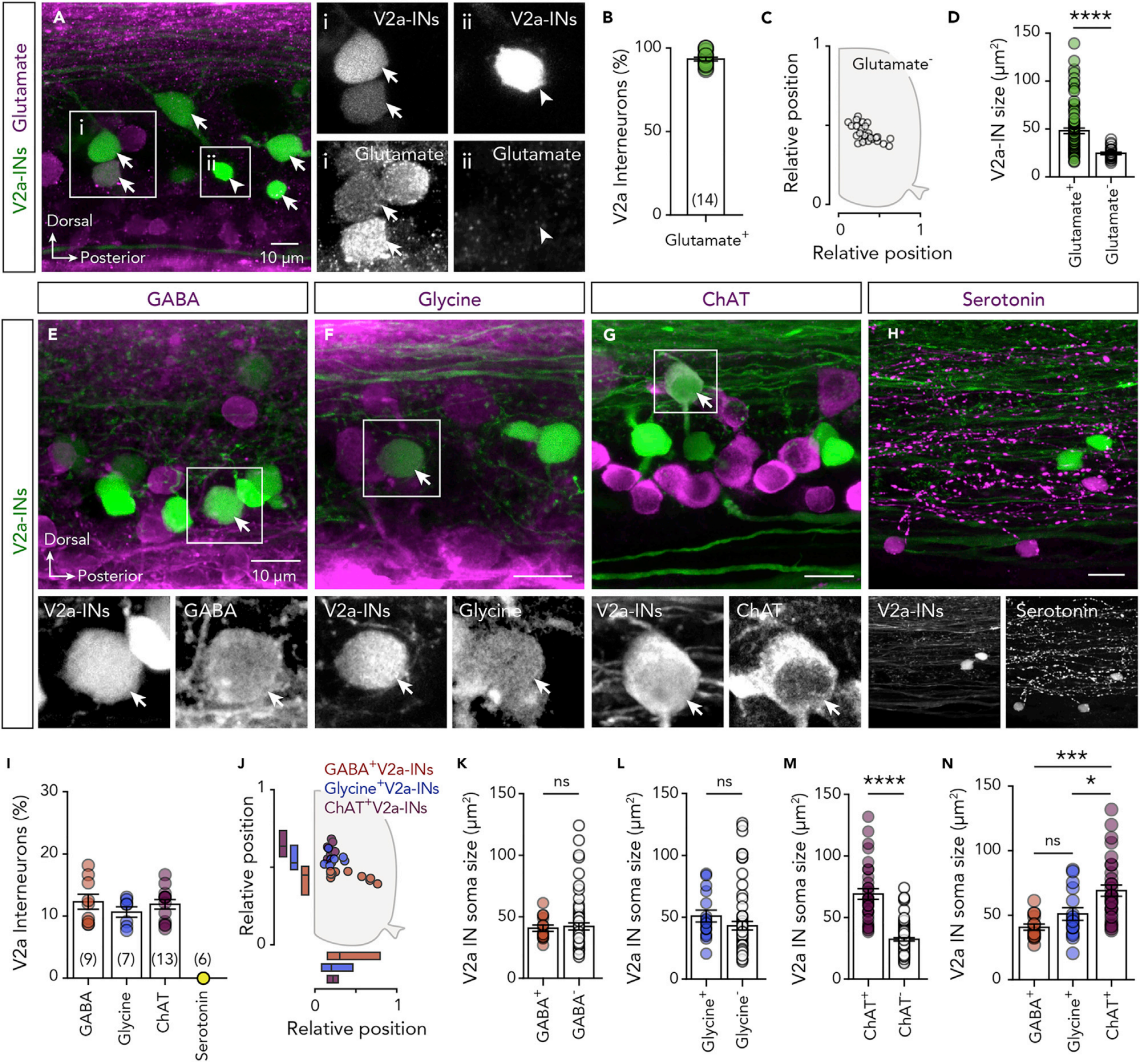
V2a Interneuron Neurotransmitter Diversity (A and B) Representative whole-mount photomicrographs and analysis showing that the vast majority, but not all, of the adult zebrafish spinal cord V2a interneurons are expressing glutamate. Arrows indicate the double-labeled neurons. Arrowheads indicate the non-glutamatergic V2a interneurons. (C) Setting positions of the glutamate^−^ (open circles) V2a interneurons in the spinal cord. (D) Plot showing the difference in soma sizes of the glutamate^+^ (green circles) and glutamate^−^ (open circles) V2a interneurons. (E-H) Whole-mount double immunolabeling between V2a interneurons with GABA, glycine, ChAT, or serotonin. In black and white are single channel images of the merged images. Arrows indicate the double-labeled neurons. (I and J) Analysis of the percentage and the topographic organization of the V2a interneurons that express GABA, glycine, or ChAT. (K–M) Quantification of the V2a interneuron soma sizes that are immune-positive and immune-negative for the GABA, glycine, or ChAT (unpaired t test: t = 10.65, df = 111, P < 0.0001). (N) Comparison of the V2a interneuron soma sizes that express GABA, glycine, or ChAT (one-way ANOVA: F_(2,58)_ = 10.44, P = 0.0001). Data are presented as mean ± SEM. *P < 0.05; ***P < 0.001; ****P < 0.0001; ns, non-significant. For related data and antibodies information, see also Figure S4 and Table S1.

To evaluate our findings and the extent of neurotransmitter co-expression and dynamics, we performed a proof-of-concept analysis focusing on one of the most well-characterized spinal interneuron populations, the V2a interneurons (Arber, 2012, Goulding, 2009, Kiehn, 2016, Kiehn, 2011). V2a interneurons are one of the most important excitatory neuronal classes for the operation of the vertebrate locomotor network (Al-Mosawie et al., 2007, Crone et al., 2008, Dougherty and Kiehn, 2010, Hayashi et al., 2018, Joshi et al., 2009, Lundfald et al., 2007, Zhong et al., 2011), as demonstrated by studies on zebrafish (Ampatzis et al., 2014, Ausborn et al., 2012, Eklöf-Ljunggren et al., 2012, Kimura et al., 2006, McLean et al., 2008, McLean and Fetcho, 2009, Menelaou et al., 2014, Song et al., 2018). In keeping with previous reports (Ampatzis et al., 2014), we detected 23.59 ± 0.503 V2a interneurons (*n* = 22 zebrafish; Figure S4B) per hemisegment in the adult zebrafish spinal cord. These interneurons were found to be distributed within the motor column (Figure S4C) and displayed variable soma sizes (Figure S4D). Detailed analysis of the neurotransmitter phenotype of the V2a interneuron population revealed that the vast majority (93.27 ± 1.116%, *n* = 17 zebrafish; Figures 5A and 5B) were glutamatergic, with occasionally (10 of 14 zebrafish) one and rarely two GFP^+^ V2a interneurons appearing as glutamate^−^. The glutamate^−^ V2a interneurons had restricted distribution (Figure 5C) and significantly smaller soma (24.61 ± 1.261 μm^2^) than those expressing glutamate (48.21 ± 2.902 μm^2^; unpaired t test: t = 4.351, df = 104, P < 0.0001, Figure 5D). Interestingly, a smaller fraction of the V2a interneurons appeared to also express GABA (12.31 ± 1.217%, *n* = 9 zebrafish; Figures 5E and 5I), glycine (10.67 ± 0.84%, *n* = 7 zebrafish; Figures 5F and 5I), or ChAT (11.9 ± 0.755%, *n* = 13 zebrafish; Figures 5G and 5I). However, none were found to express serotonin (*n* = 6 zebrafish; Figures 5H and 5I). Moreover, the GABA^+^, glycine^+^, and ChAT^+^ V2a interneurons had distinct topographic distribution patterns (Figure 5J) and soma sizes (Figures 5K–5N), strongly suggesting that they may constitute different subpopulations of the glutamatergic population. Finally, we sought to determine whether the glutamatergic V2a interneurons could co-transmit these additional neurotransmitters by performing immunohistochemistry and *in situ* hybridization experiments to investigate their ability to produce the vesicular transporters for GABA and glycine (vGAT) and for acetylcholine (vAChT) (Figure S4). We detected vAChT, vGAT, and the glycinergic transporter (GlyT2) in presynaptic terminals (SV2^+^) of the V2a interneurons (GFP^+^, Figure S4E). In addition, vGAT or vAChT mRNAs were detected in a small proportion of the V2a interneurons by *in situ* hybridization (Figure S4F). These findings confirm our immunohistochemical observations (Figures 5E–5N) and suggest that the V2a interneurons' functional role in the organization and operation of the spinal cord networks controlling animals' movements is more complex than previously recognized.

## Discussion

We have conducted the first comprehensive classification of adult zebrafish neurons in a whole spinal cord hemisegment, revealing the total number of neurons, their sizes, the transmitter phenotypes they express, their setting positions, and their projection patterns. We have also established the extent of co-expression of the main classical neurotransmitters in spinal cord neurons, suggesting that the neurons' chemical and anatomical organization is much more complex than previously recognized. Neuronal maps like that presented here, which describe distinct structural and biochemical features, provide essential guidance for future studies on the nervous system's development and function. Cell-type-specific neurotransmitter classifications of spinal neurons will enable further functional analyses of the diverse but stereotypic neuron populations that generate and gate sensory and motor functions to control animal movements.

Signal transmission in neuronal networks involves the release of neurotransmitters that bind specifically to membrane receptors on target neurons to mediate basic and complex biological functions. Since the identity of the neurotransmitters that a neuron synthesizes and releases is an important aspect of its differentiation fate, it is essential to understand the genetic programs that specify an individual neuron's type and transmitter expression. The genetic programs that specify the spinal cord neuronal populations are well defined (Alaynick et al., 2011, Arber, 2012, Goulding, 2009, Jessell, 2000, Kiehn, 2016), but our understanding of neurotransmission within these neuronal classes is limited. Among the neurotransmitters of the nervous system, glutamate, GABA, glycine, acetylcholine, and serotonin are the most well studied in the spinal cords of vertebrates (Alvarez et al., 2005, Antal et al., 1994, Brodin et al., 1990, Mahmood et al., 2009, Phelps et al., 1990, Pombal et al., 2001, Restrepo et al., 2009, Sueiro et al., 2004, Wéber et al., 2007), including zebrafish (Barreiro-Iglesias et al., 2013, Bradley et al., 2010, Böhm et al., 2016, Higashijima et al., 2004a, Higashijima et al., 2004b, Liao and Fetcho, 2008, McLean and Fetcho, 2004). Several spinal interneuron types have been described in the developing zebrafish spinal cord based on their discrete morphological features (Bernhardt et al., 1990, Bernhardt et al., 1992, Hale et al., 2001), which have been linked to specific neurotransmitter identities (Higashijima et al., 2004a, Higashijima et al., 2004b). These associations imply that most descending projecting interneurons express glutamate, whereas most ascending projecting neurons express GABA and/or glycine. This reinforces the notion that the principal descending input in the spinal cord is excitatory and the main ascending input is inhibitory. However, our tracing and immunodetection experiments suggest that similar numbers of inhibitory and excitatory neurons project to the rostral and caudal parts of the spinal cord and the vast majority of these neurons are glutamatergic.

Our results firmly establish that many spinal cord neurons (∼15%; approximately 80–90 neurons) exhibit multiple neurotransmitter phenotypes. One classical view in neuroscience is that neurons have the ability to produce, store, and release one type of neurotransmitter, a misinterpreted concept of Dale's principle (Eccles et al., 1954), that a neuron releases the same neurotransmitter(s) from all of its synapses. This view introduced a strongly reductionist approach to nervous system complexity by assigning each neuron to one of three functional classes (excitatory, inhibitory, or modulatory). Recently, however, several findings have complicated this simple characterization: there is growing evidence that neuronal populations in vertebrate and invertebrate nervous systems use multiple transmitter systems simultaneously. The possibility that neurons may release multiple neurotransmitters was first suggested by Burnstock (1976). Subsequent anatomical studies demonstrated the co-localization of multiple transmitters in single neurons (Hökfelt et al., 1977, Hökfelt et al., 1987, Hökfelt et al., 1998), and functional investigations have shown that many neuronal subtypes can store and release multiple neurotransmitters simultaneously (Granger et al., 2016, Hnasko and Edwards, 2012, Noh et al., 2010, Seal and Edwards, 2006, Vaaga et al., 2014). Nowadays, the concept of neurotransmitter co-release by single neurons is well accepted, and many, if not most, neurons are understood to use multiple transmission. However, the prevalence and physiological roles of co-transmission remain poorly understood, as is the synaptic circuitry involved.

The adult zebrafish spinal cord neurotransmitter atlas presented here is an essential resource for identifying currently unknown subpopulations of spinal neurons and for future comparative studies on spinal circuit organization. Our anatomical mapping revealed a population of adult spinal neurons expressing both GABA and glycine, as previously demonstrated during zebrafish development (Higashijima et al., 2004a). It is well established that many neurons in the vertebrate spinal cord co-express and release these inhibitory neurotransmitters (Alvarez and Fyffe, 2007, Chery and de Koninck, 1999, Geiman et al., 2002, Taal and Holstege, 1994, Todd et al., 1996, Svensson et al., 2018). Moreover, in keeping with our observations here, it is well established that the vertebrate cholinergic spinal neurons (motoneurons) can co-express and co-release glutamate along with acetylcholine (Bertuzzi et al., 2018, Meister et al., 1993, Mentis et al., 2005, Nishimaru et al., 2005). Interestingly, we also found that spinal cord neurons exhibit extensive co-expression of glutamate and GABA, two neurotransmitters with opposing functions. Although we did not investigate the release of these transmitters in this work, the possible co-release of glutamate and GABA from single nerve terminals in the brain has been demonstrated extensively (Beltrán and Gutiérrez, 2012, Galván and Gutiérrez, 2017, Noh et al., 2010, Root et al., 2014, Shabel et al., 2014, Yoo et al., 2016). Our findings support the existence of multi-transmitter neurons in the zebrafish spinal cord, as was already established in the lamprey spinal cord (Fernández-López et al., 2012) and the mammalian brain (Granger et al., 2017, Tritsch et al., 2016). However, the co-expression and co-release of these diverse transmitter combinations in mammalian spinal neurons has yet to be confirmed. Since the spinal cord is an evolutionarily conserved region of the central nervous system (Arber, 2012, Grillner, 2003, Grillner and Jessell, 2009), our results are probably relevant to organisms of higher phylogenetic order, including mammals. Based on this evolutionary perspective, we suggest that the diversity and complexity of zebrafish spinal neurons is likely to be echoed on larger scales in mammalian spinal systems, enabling better control of far more complex motor behaviors.

Our analysis also shows that the V2a interneurons form a functionally heterogeneous class of neurons that co-express GABA, glycine, or ChAT in addition to glutamate. Although several previous studies on the anatomical and functional organization of the V2a interneurons neglected the possibility that they might co-express and potentially co-release neurotransmitters other than glutamate (Ampatzis et al., 2014, Ausborn et al., 2012, Dougherty and Kiehn, 2010), previous attempts were made for the characterization of their neurotransmitter phenotype (Lundfald et al., 2007). In the developing mammalian spinal cord, most (∼80%) of the V2a interneurons were observed to be glutamatergic, and a small fraction (∼5%) to be putative glycinergic, but none GABAergic (*GAD*_*67*_*:GFP*^+^; Lundfald et al., 2007). However, these findings cannot exclude the possibility that GABAergic mammalian V2a interneurons exist as they can alternatively use the GAD_65_ as glutamate decarboxylase, which is present in a different set of neurons within the central nervous system (Ma et al., 1994, Feldblum et al., 1995, Lee et al., 2011). Furthermore, in line with our data presented here, a recent single-cell transcriptome analysis of the mammalian spinal cord neurons revealed the presence of the cholinergic vesicular transporter (vAChT), of the vIAAT and the GlyT2 in the V2a interneuron population (Delile et al., 2019). Together this demonstrated diversity of the V2a interneurons can reflect their functional heterogeneity that has been observed before in both zebrafish (Ampatzis et al., 2014, Ausborn et al., 2012, Song et al., 2018) and mice (Al-Mosawie et al., 2007, Zhong et al., 2011). In particular, the V2a interneurons in adult zebrafish form three discrete functional subpopulations that are incrementally recruited at different speeds of locomotion, and their recruitment pattern is not topographically organized (Ampatzis et al., 2014, Ausborn et al., 2012, Song et al., 2018). Although our findings indicate that a small fraction of the V2a interneurons can co-express other classical neurotransmitters in addition to glutamate, it seems very unlikely that these other neurotransmitters are released to control spinal motoneuron activity (Ampatzis et al., 2014, Song et al., 2016, Song et al., 2018). It seems more likely that these additional neurotransmitters mediate the neuronal interplay needed for the precise operation of the central pattern generators and may also contribute to the establishment of the necessary rostro-caudal delay.

### Limitations of the Study

Although the immunodetection and *in situ* hybridization methods have considerable advantages enabling the detailed analysis of the transmitter phenotypes of the spinal neurons, several key limitations remain. First, the immunodetection of the transmitter neural phenotypes is constrained to cell somata. Thus, future functional validation of the co-transmission remains to be determined, in particular, to functionally validate the glutamatergic nature of the spinal cord neurons, as high concentrations of glutamate could exist in metabolically active cells (Storm-Mathisen et al., 1986, Zhang et al., 1991). Second, the fluorescent microscopic analysis is limited to the number of fluorescent probes that are currently available. Thus, our study may be considered conservative and underestimate the full neurotransmitter complexity that exists in the vertebrate spinal neurons. A complete and accurate single-cell transcriptomic analysis will help to overcome this critical limitation. Third, it is essential to be aware that the study here considered the neurotransmitter phenotypes as fixed. However, neurons can dynamically change their neurotransmitter phenotypes, under both physiological and pathophysiological conditions (Black et al., 1984, Dulcis et al., 2013, Dulcis et al., 2017, Spitzer, 2015, Spitzer, 2017, Bertuzzi et al., 2018).

## Methods

All methods can be found in the accompanying Transparent Methods supplemental file.

## Supplemental Information

Document S1. Transparent Methods, Figures S1–S5, and Table S1
