# Supplementary material for: Large-Scale Analysis of the Diversity and Complexity of the Adult Spinal Cord Neurotransmitter Typology
Source: iScience. 2019 Sep 10;19:1189–201. doi: 10.1016/j.isci.2019.09.010 (PMC6831849; doi:10.1016/j.isci.2019.09.010)
Supplement: Document S1. Transparent Methods, Figures S1–S5, and Table S1 [file mmc1.pdf]

ISCI, Volume *19*

Supplemental Information

Large-Scale Analysis of the Diversity and Complexity of the Adult Spinal Cord Neurotransmitter Typology

Andrea Pedroni and Konstantinos Ampatzis

Supplemental Information

Large scale analysis of the diversity and complexity of the adult spinal cord neurotransmitter typology

Andrea Pedroni and Konstantinos Ampatzis

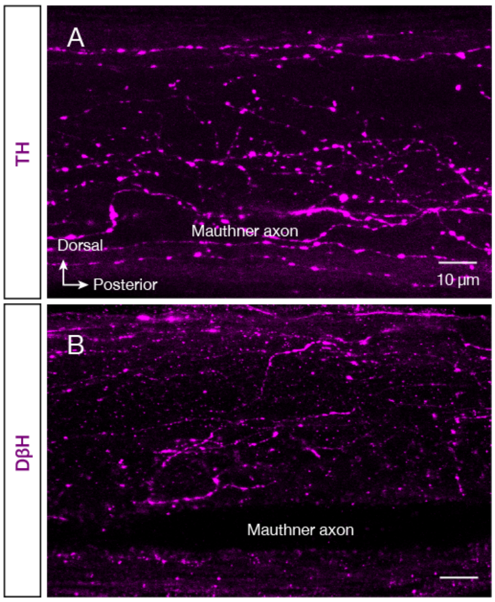

Figure S1. Lack of spinal dopaminergic and noradrenergic neurons, Related to Figure 2.
(A-B) Representative whole mount confocal images showing that only dopaminergic (TH^+) and noradrenergic ($D\beta H^+$) neuronal processes are detectable in the adult zebrafish spinal cord.

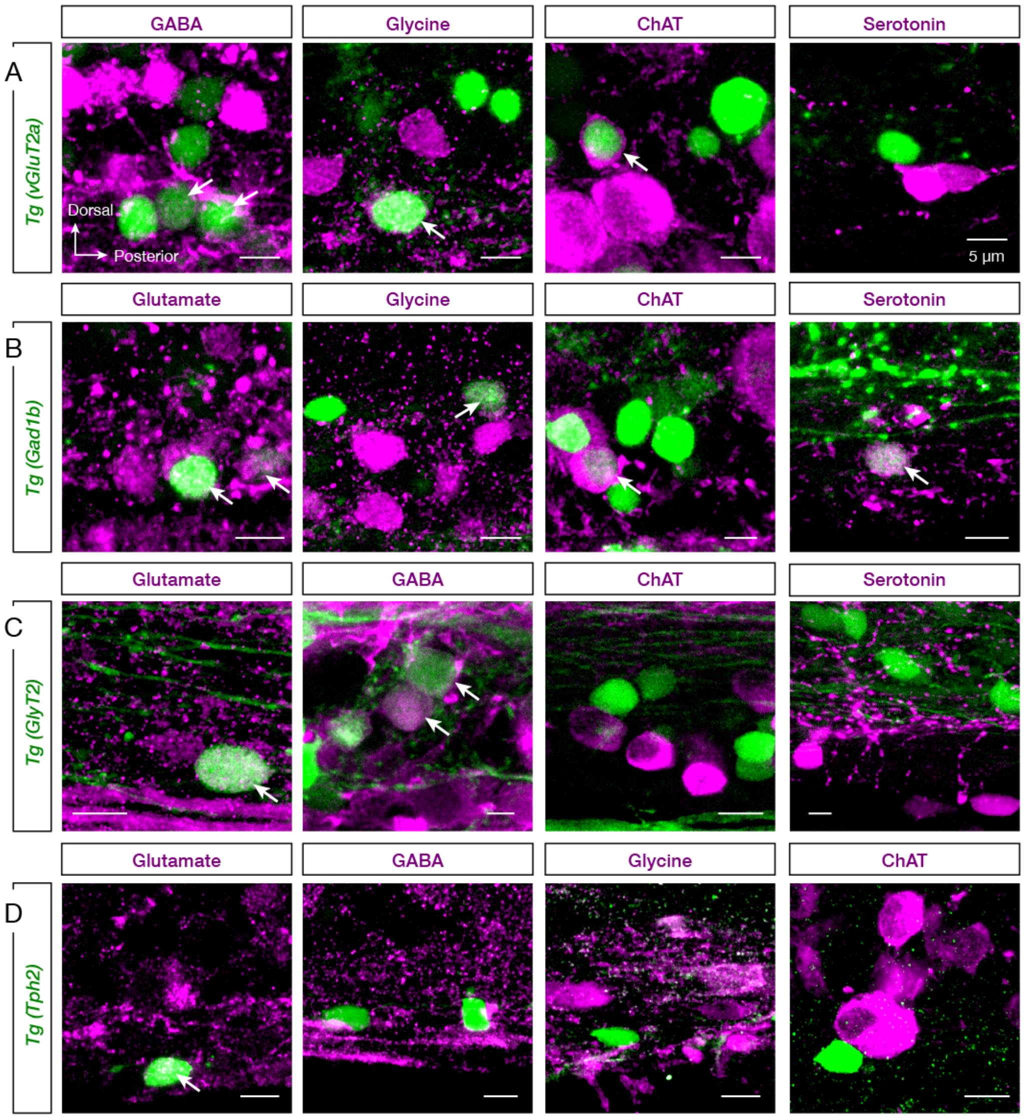

Figure S2. The presence of co-expressing neurons in transgenic animal lines, Related to Figure 4. (A-D) Representative whole mount images from transgenic (*GlyT2*, *vGluT2a*, *Gad1b*, *Tph2*; green) adult zebrafish spinal cord preparations, immunolabeled for glutamate, GABA, glycine, ChAT and serotonin (magenta). Arrows indicate the double labeled cells.

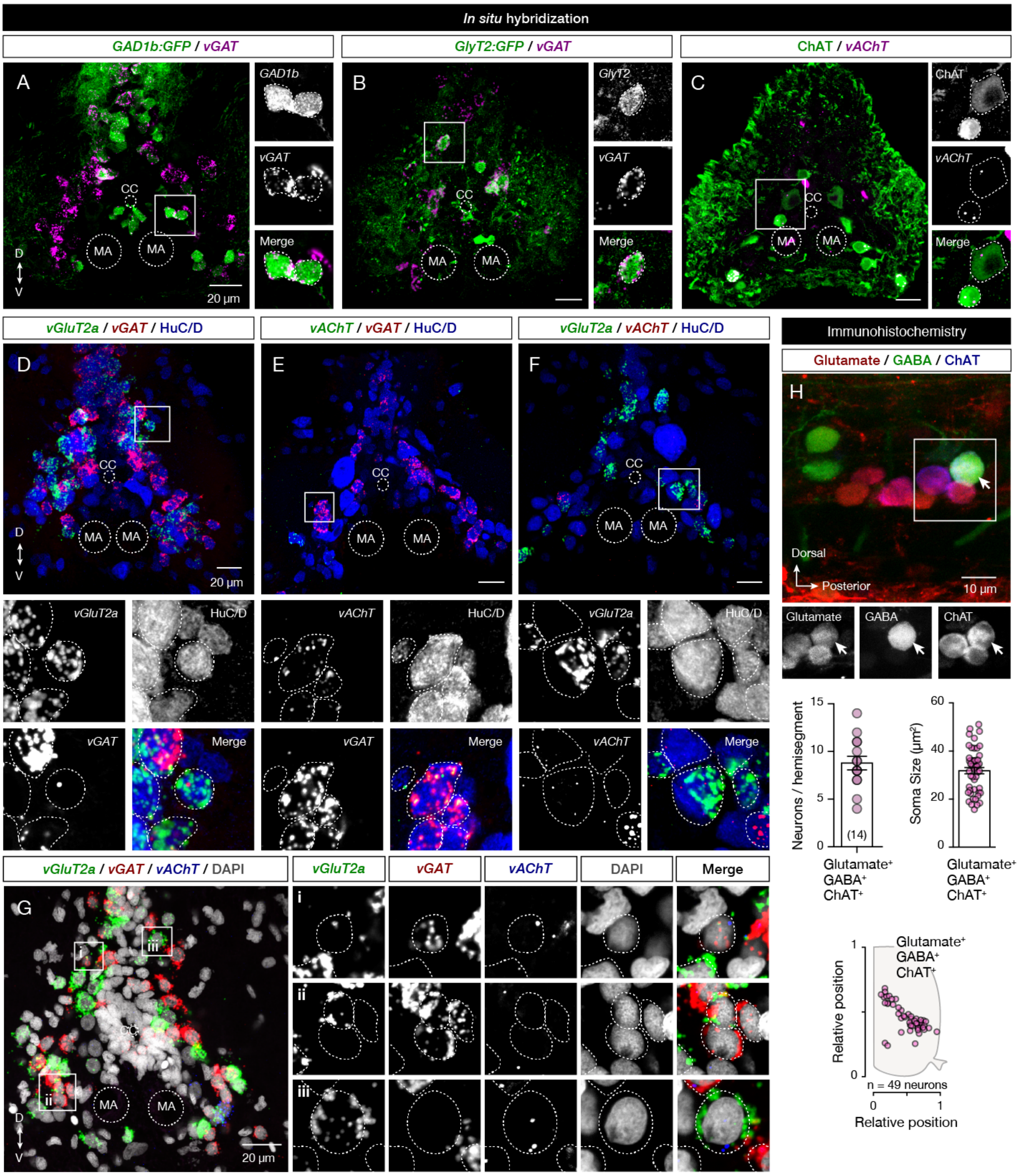

Figure S3. ***In situ* detection of the neurotransmitter's vesicular transporters, Related to Figure 4.**
(A-B) Confocal photomicrographs of transversal sections of the adult zebrafish spinal cord showing the presence of the *vGAT* mRNA in all the putative GABAergic (*GAD1b*) and Glycinergic (*GlyT2*) neurons.
(C) *In situ* hybridization reveals a small number of the vesicular acetylcholine transporter (*vAChT*) mRNA in all the cholinergic spinal neurons ($ChAT^{+}$).
(D-F) Co-localization of different vesicular transporter mRNAs in adult zebrafish spinal cord.
(G) Representative *in situ* hybridization for all the vesicular transporters (*vGluT2a*, *vGAT* and *vAChT*) mRNA showing a co-localization in the adult zebrafish spinal cord neurons.
(H) Triple immunolabeling confirms the existence of multi-expressing, glutamate (red), GABA (green) and ChAT (blue) neurons. Arrow indicates the triple labeled neuron. Quantification of the number and spatial distribution of cholinergic neurons co-expressing simultaneously glutamate and GABA in the adult zebrafish spinal cord hemisegment (segment 15). Quantification of the $ChAT^{+}Glutamate^{+}GABA^{+}$ neurons soma size.
Dotted lines represent the borders of the neurons. Data are presented as mean ± SEM. CC, central canal; MA, Mauthner axon.

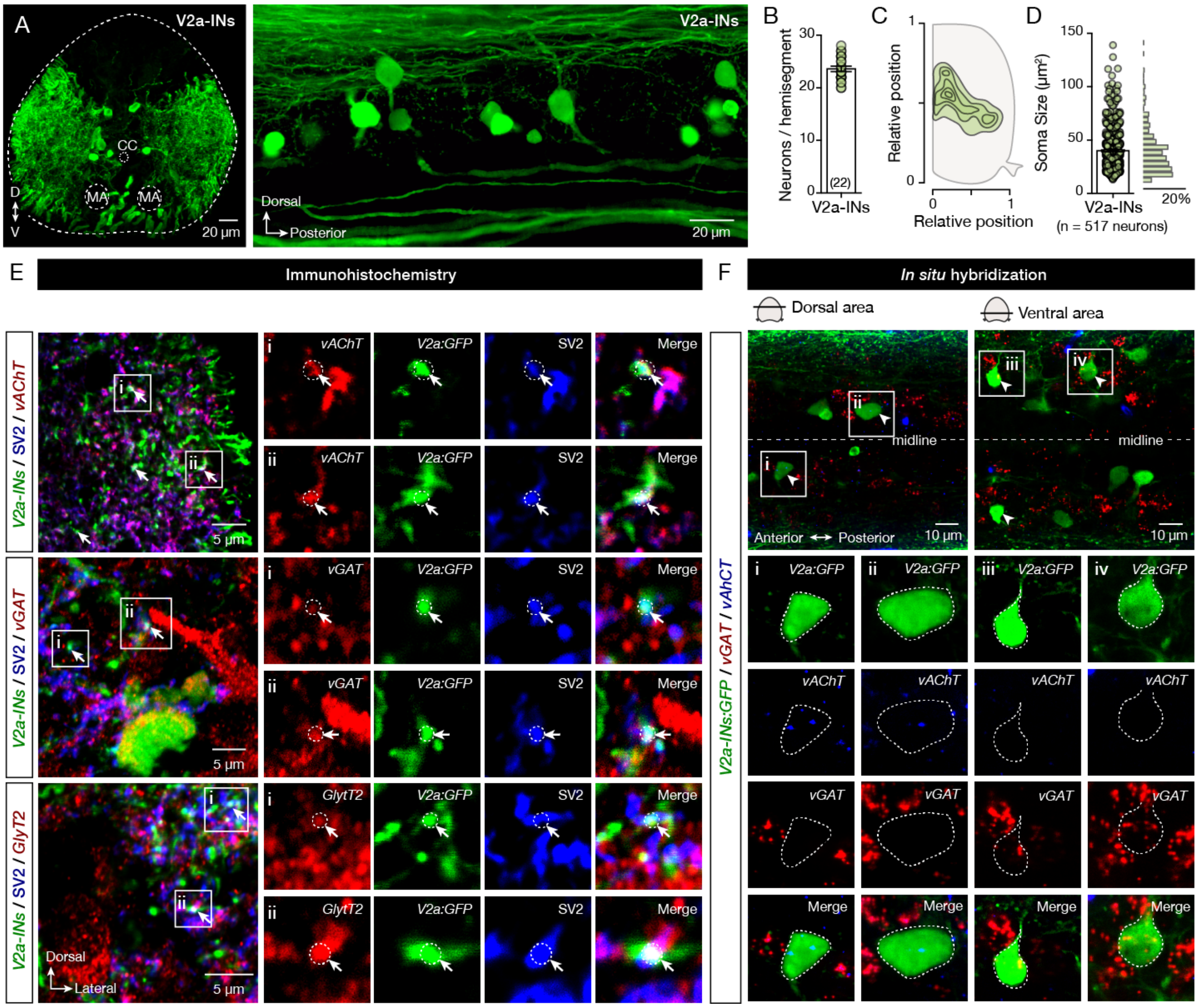

Figure S4. V2a interneuron analysis, Related to Figure 5.
(A) Transverse section and whole mount adult zebrafish spinal cord showing the distribution of the V2a interneuron population.
(B) Quantification of the number of V2a interneurons in adult spinal cord hemisegment (segment 15).
(C) Spatial distribution of the V2a interneurons with the medio-lateral and dorso-ventral density plot.
(D) Quantification and distribution analysis of the V2a interneuron soma sizes (*n* = 517 neurons).
(E) Confocal photomicrographs of transversal sections of the adult zebrafish spinal cord showing the colocalization of the presynaptic V2a interneuron terminals ($GFP^+/SV2^+$) with the vesicular acetylcholine transporter (vAChT), the vesicular GABA and glycine transporter (vGAT) or with the glycinergic transporter (GlyT2). Arrows indicate the triple co-localization.
(F) Whole mount *in situ* hybridization reveals the co-existence of the different vesicular neurotransmitter transporter mRNAs (*vAChT* or *vGAT*) in the V2a (GFP^+) interneurons in the expected relative locations (dorsal, ventral) within the adult zebrafish spinal cord. Arrowheads indicate the double labeled neurons.
Data are presented as mean ± SEM. CC, central canal; MA, Mauthner axon.

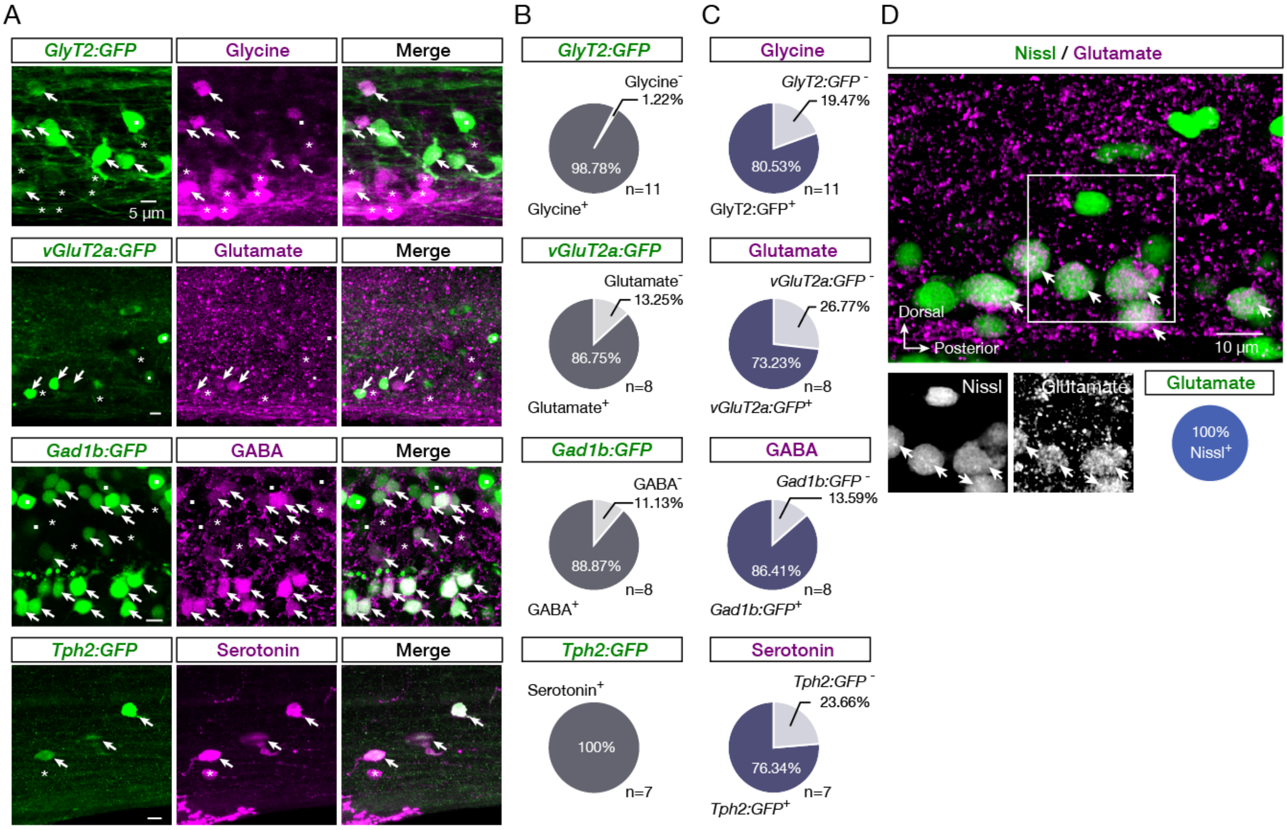

Figure S5. Transgenic lines capture part of the labeled neuronal populations, Related to Figure 2.
(A) Representative immunofluorescent whole-mount images showing immunostained neurons in the spinal cord of transgenic zebrafish with glycinergic (*GlyT2*), glutamatergic (*vGluT2a*), GABAergic (*Gad1b*) or serotonergic (*Tph2*) neurons genetically labelled (green). Asterisks denote the immunopositive neurons that do not express GFP, dots indicate the GFP expressing neurons that are not immunoreactive and arrows identify double labelled neurons.
(B) Quantification of the percentage of immunolabeled neurons that express GFP.
(C) Quantification of the percentage of GFP^+ neurons that are labeled with antibodies.
(D) Representative whole mount image of adult zebrafish spinal cord, immunolabeled for glutamate (magenta) with neurons identified by Nissl staining (green). Arrows indicate the double labeled cells.

TABLE S1. Antibodies Used[1], Related to Figures 1-5.

Antigen	Host	Source	Code	Dilution
Primary				
ChAT	Goat	Millipore	AB144P; RRID: AB_2079751	1:150
DβH	Rabbit	Millipore	AB1538; RRID: AB_90751	1:250
Elav3+4 (HuC/D)	Rabbit	GeneTex	GTX128365; RRID: AB_	1:500
GABA	Rabbit	Sigma	A2052; RRID: AB_477652	1:2000
GABA	Mouse	From Prof. P. Streit; RRID: AB_2314450		1:700
Glutamate	Rabbit	Sigma	G6642; RRID: AB_259946	1:6000
Glycine	Rat	ImmunoSolutions	IG1002; RRID: AB_10013222	1:1000
Serotonin	Rabbit	Sigma	S5545; RRID: AB_477522	1:4000
Serotonin	Guinea Pig	From Prof. A. Verhofstadt		1:2500
TH	Mouse	Millipore	MAB318; RRID: AB_2201528	1:800
GFP	Chicken	Abcam	AB13970; RRID: AB_300798	1:500
vGAT	Rabbit	Synaptic Systems	131 002; RRID: AB_887871	1:300
GlyT2	Rabbit	Alomone labs	AGT-012; RRID: AB_11121049	1:200
vAChT	Guinea Pig	Millipore	AB1588; RRID: AB_11214110	1:1000
SV2	Mouse	DSHB	SV2; RRID: AB_2315387	1:200
Secondary				
Chicken IgY-488	Goat	ThermoFisher	A-11039; RRID: AB_2534096	1:500
Chicken IgY-FITC	Rabbit	ThermoFisher	SA1-9511; RRID: AB_1075130	1:500
Goat IgG-568	Donkey	ThermoFisher	A-11057; RRID: AB_2534104	1:500
Goat IgG-488	Donkey	ThermoFisher	A-11055; RRID: AB_2534102	1:500
Goat IgG-647	Donkey	ThermoFisher	A-21447; RRID: AB_2535864	1:500
Guinea Pig IgG-568	Goat	ThermoFisher	A-11075; RRID: AB_2534119	1:500
Mouse IgG-647	Donkey	ThermoFisher	A-31571; RRID: AB_162542	1:500
Mouse IgG-568	Goat	ThermoFisher	A-11004; RRID: AB_2534072	1:500
Mouse IgG-488	Donkey	ThermoFisher	A-21202; RRID: AB_141607	1:500
Rabbit IgG-488	Donkey	ThermoFisher	A-21206; RRID: AB_2535792	1:500
Rabbit IgG-647	Donkey	ThermoFisher	A-31573; RRID: AB_2536183	1:500
Rabbit IgG-568	Donkey	ThermoFisher	A-10042; RRID: AB_2534017	1:500
Rat IgG-550	Donkey	ThermoFisher	SA5-10027; RRID: AB_2556607	1:500
Rat IgG-568	Goat	ThermoFisher	A-11077; RRID: AB_2534121	1:500

[1]ChAT, choline-acetyltransferase; DβH, dopamine beta-hydroxylase; GABA, γ-aminobutyric acid; GFP, green fluorescent protein; GlyT2, glycine transporter 2; TH, tyrosine hydroxylase; SV2, synaptic vesicle glycoprotein 2A; vAChT, vesicular acetylcholine transporter; vGAT, vesicular GABA transporter.

Transparent methods

Experimental animals

All animals were raised and kept in a core facility at the Karolinska Institute according to established procedures. Adult zebrafish (*Danio rerio*; *n* = 635; 9-10 weeks old; length: 16-19 mm; weight: 0.03-0.05 g) wild type (AB/Tübingen), and *Tg*(*vGluT2a:GFP; vGluT2a:DsRed; Gad1b:GFP; Gad1b:DsRed; GlyT2:GFP; Tph2:Gal4^{y228}/UAS:GFP; Chx10:GFP^{nns1}*) lines of either sex (males and females) where used in this study. All experimental protocols were approved by the local Animal Research Ethical Committee, Stockholm (Ethical permit no. 9248-2017) and were performed in accordance with EU guidelines for the care and use of laboratory animals (86/609/CEE). All efforts were made to utilize only the minimum number of experimental animals necessary to produce reliable scientific data.

Immunohistochemistry

All animals were deeply anesthetized with tricaine methane sulfonate (MS-222, Sigma-Aldrich, E10521). The spinal cords were then extracted and fixed in 4% paraformaldehyde (PFA) and 5% saturated picric acid (Sigma-Aldrich, P6744) in phosphate buffer saline (PBS) (0.01M; pH = 7.4, Santa Cruz Biotech., CAS30525-89-4) at 4°C for 2-14h. We performed immunolabeling in both whole mount spinal cords and in cryosections. For cryosections, the tissue was removed carefully and cryoprotected overnight in 30% (w/v) sucrose in PBS at 4°C, embedded in OCT Cryomount (Histolab, 45830), rapidly frozen in dry-ice-cooled isopentane (2-methylbutane; Sigma-Aldrich, 277258) at approximately –35°C, and stored at –80°C until use. Transverse coronal plane cryosections (thickness: 25 µm) of the tissue were collected and processed for immunohistochemistry. In all cases (whole mount and cryosections) the tissue was washed three times for 5 min in PBS (0.01M; pH = 7.4, Santa Cruz Biotech., SC24946). Nonspecific protein binding sites were blocked with 4% normal donkey serum (NDS; Sigma-Aldrich, D9663) with 1% bovine serum albumin (BSA; Sigma-Aldrich, A2153) and 1% Triton X-100 (Sigma-Aldrich, T8787) in PBS for 1 h at room temperature (RT). Primary antibodies (Table S1) were diluted in 1% of blocking solution and applied for 1-3 days at 4°C. After thorough buffer rinses the tissues were then incubated with the appropriate secondary antibodies (Table S1) diluted 1:500 or with streptavidin conjugated to Alexa Fluor 488 (1:500, ThermoFisher, S32354) Alexa Fluor 555 (1:500, ThermoFisher, S32355) or Alexa Fluor 647 (1:500, ThermoFisher, S32357) in 1% Triton X-100 (Sigma-Aldrich, T8787) in PBS overnight at 4°C. Afterwards, the tissue was thoroughly rinsed in PBS and cover-slipped with fluorescent hard medium (VectorLabs; H-1400) on gelatine-coated microscope slides.

For the anti-GABA antisera raised in mouse (Table S1), the tissue was fixed in 4% paraformaldehyde (PFA) containing 0.2% glutaraldehyde (Sigma-Aldrich, G5882) and 5% saturated picric acid in phosphate buffer saline (PBS; 0.01M; pH = 7.4) at 4°C for 10h. Finally, dual and triple neurotransmitter antibody stainings were performed in a sequential manner.

Antibody specificity

The antisera in this study have been widely and successfully used previously in zebrafish to identify and describe the transmitter phenotype of neurons (anti-ChAT: Berg et al., 2018; Bertuzzi and Ampatzis 2018; Bertuzzi et al., 2018; Clemente et al., 2004; Mueller et al., 2004; Moly et al., 2014; Ohnmacht et al., 2016; Reimer et al., 2008; anti-GABA: Berg et al., 2018; Djenoune et al., 2017; Higashijima et al., 2004; Moly et al., 2014; Montgomery et al., 2016; Mueller et al., 2006; anti-Glycine: Berg et al., 2018; Moly et al., 2014; anti-Serotonin: Berg et al., 2018; Kuscha et al., 2012; McPherson et al., 2016; Montgomery et al., 2016; anti-TH: Ampatzis et al., 2008; Ampatzis and Dermon 2010; Rink and Wullimann, 2004; anti-DβH: Ampatzis et al., 2008), and the green fluorescent protein (anti-GFP: Barreiro-Iglesias et al., 2013; Böhm et al., 2016; Kuscha et al., 2012).

To evaluate the antibody specificity, adjacent sections or additional whole mount spinal cords were used in the absence of either primary or secondary antibody. In all cases no residual immunolabeling was detected. In addition, pre-incubation of the neurotransmitter antibodies used in this study with their corresponding antigen (100 µM - 400 µM; GABA (Sigma-Aldrich, A2129), glutamate (Sigma-Aldrich, G3291), glycine (Sigma-Aldrich, G6761), and serotonin (Sigma-Aldrich, 14927) for 1h at RT, eliminated any immunoreactivity.

To further confirm that our antibody immunodetections faithfully represented the neurotransmitter expression of spinal cord neurons, we performed a series of control experiments using transgenic animal lines (*vGluT2a, Gad1b, GlyT2, and Tph2*) combined with immunolabeling. We found that the vast majority or all of the neurons expressing the green fluorescent protein (GFP or DsRed) in either the vesicular

glutamate transporter *vGluT2a*, a marker for glutamatergic excitatory neurons, the GABAergic inhibitory neuron marker *Gad1b*, the glycinergic neuronal marker *GlyT2* or the *Tph2* promoter for serotonergic neurons were immunolabeled with the anti-glutamate, anti-GABA, anti-glycine and anti-serotonin antibodies, respectively (Figure S5A-S5B). We also observed that immunostaining revealed more neurons than those expressing GFP (Glutamate^{+}*vGluT2a*$^{-}$: 26.77 ± 1.527%, *n* = 8; GABA^{+}*Gad1b*$^{-}$: 13.59 ± 1.134%, *n* = 8; Glycine^{+}*GlyT2*$^{-}$: 19.47 ± 0.449%, *n* = 11; Serotonin^{+}*Tph2*$^{-}$: 23.66 ± 1.723%, *n* = 7; Figure S5C). These findings suggest a differential expression of the glutamatergic vesicular transporter vGluT2a from the vGluT1 (in zebrafish spinal motoneurons, Bertuzzi et al., 2018) and/or vGlut3 glutamatergic neurons. Similarly, these findings propose the existence of GABAergic neurons that use the alternative GAD1a and/or GAD2 as a glutamate decarboxycase enzyme and finally the presence of GlyT1 glycinergic neurons. This observed discrepancy between the immunolabelling and the reporter zebrafish lines signify that although the transgenic zebrafish lines consistently label the neurons of the expected neurotransmitter phenotype, they do not mark all neurons within a population. In addition, we used the transgenic animal lines as described above combined with single immunostaining for glutamate, GABA, glycine, ChAT, and serotonin. We observed, that similar to dual immunodetections, the vast majority of the different combinations of co-expression in spinal cord neurons (Figure S2). Regarding the specificity and reliability of the anti-glutamate immunodetection, we performed additional experiments to evaluate whether the glutamate^{+} cells are actually excitatory neurons, as glial cells can also store and release glutamate (Angulo et al., 2004; Hamilton and Attwell, 2010). Double staining with the anti-glutamate antisera and the NeuroTrace 500/525 Green fluorescent Nissl stain (ThermoFisher, N21480) showed that all the immunolabeled are indeed neurons (Figure S5D).

RNAscope *in situ* hybridization

RNAscope *In situ* hybridization experiments shown in Figures S3 and S4, were performed according to manufacturer's instructions in cryosections and in whole mound spinal cord preparations. The vesicular neurotransmitter transporter mRNAs were detected using RNAscope (Advanced Cell Diagnostics) zebrafish designed target probes (*slc17a6b,* also known as *vGluT2a,* Cat# 300031; *slc18a3b*, also known as *vAChT*, Cat# 300031-C2; *slc32a1,* also known as *vGAT* or *vIAAT*, Cat# 300031-C3). Following the *in situ* hybridization, cell nuclei and cell body were revealed using respectively the DAPI staining and anti-Elav 3+4 (HuC/D) immunodetection. In V2a interneuron (*Chx10:GFP*^{nns1}), glutamate decarboxylase 1b transgenic (*GAD1b:GFP*), and in glycine transporter 2 (*GlyT2:GFP*) transgenic zebrafish line, the tissue was further processed for immunohistochemical detection of the GFP in the same way as in all immunodetections (see above, "Immunohistochemistry" section). The tissue was processed and mounted on Thermo Scientific™Superfrost Plus™Adhesion microscope slides and cover-slipped with anti-fade fluorescent mounting medium (Vectashield Hard Set, VectorLabs; H-1400).

Ascending and descending neuron labeling

Zebrafish (*n* = 70) of either sex were anesthetized in 0.03% tricaine methane sulfonate (MS-222, Sigma-Aldrich, E10521). Retrograde labeling of ascending and descending spinal cord neurons located in the spinal segment 15 was performed using dye injections with biotinylated dextran (3000 MW; ThermoFisher, D7135) into segments 10 or 20 respectively. Afterwards, all animals were kept alive for at least 24h to allow retrograde transport of the tracer. Afterwards, all animals were deeply anesthetized with 0.1% MS-222. The spinal cords were dissected and fixed in 4% paraformaldehyde (PFA) and 5% saturated picric acid (Sigma-Aldrich, P6744) in phosphate buffer saline (PBS; 0.01M, pH = 7.4; Santa Cruz Biotech., CAS30525-89-4) at 4°C for 2-14h. The tissue was then washed extensively with PBS and incubated in streptavidin conjugated to Alexa Fluor 488 (dilution 1:500, ThermoFisher, S32354), Alexa Fluor 555 (1:500, ThermoFisher, S32355) or Alexa Fluor 647 (dilution 1:500, ThermoFisher, S32357) overnight at 4°C. Primary and secondary antibodies were applied as described before (see Experimental procedures, Immunohistochemistry section). After thorough buffer rinses the tissue was mounted on gelatine-coated microscope slides and cover-slipped with anti-fade fluorescent mounting medium (Vectashield Hard Set, VectorLabs; H-1400).

Analysis

Imaging was carried out on a laser scanning confocal microscope (LSM 800, Zeiss) using the 40x objective. For the whole mount preparations, the whole hemisegment of the spinal cord (from lateral side to medial area of the central canal) was scanned generating a z-stack (z-step size = 0.5 – 1 μm). Cell counting was performed in hemisegment 15 of the adult zebrafish spinal cord (in whole mount

preparations). The relative position of the somata of the neurons within the spinal cord, was calculated in whole mount preparations, using the lateral, dorsal, and ventral edges of the spinal cord as well as the central canal as landmarks. The relative position and soma sizes were measured using ImageJ (Schneider et al., 2012, NIH). For better visualization of our data, most of the whole mount images presented here were obtained by merging a subset of the original z-stack, showing the maximal intensity projections for each group. Analysis of the presynaptic terminals was performed of single plane confocal images taken using the 40x oil immerse objective. Colocalizing spots were detected by visual identification of structures whose color reflects the combined contribution of two or more antibodies in the merged image. In addition, analysis of fluorescent intensities for each channel was performed in Imagej software along single lines crossing the structures of interest (intensity spatial profile).
For the *In situ* hybridizations in cryosections (thickness: 25 μm) focal planes with homogeneous and reliable staining for each probe were taken generating a z-stack (z-stack 3-5 μm, z-step size < 1 μm). Only cells with visible $DAPI^+$ nuclei falling within the considered focal planes were examined for the presence of mRNA in the merged images. For the whole mount preparations, larger volumes (30 - 60 μm) of the spinal cord from lateral and from dorsal side ware scanned generating a z-stack (z-step size = 0.5 – 1 μm). As for the cryosections, only small subsets of the original stack (1-4 μm), with homogeneous and reliable staining and $DAPI^+$ nuclei falling within the considered focal planes, were examined for the presence of mRNA in the merged images. All the images were processed in Imagej software.
All figures and graphs were prepared with Adobe Photoshop and Adobe Illustrator (Adobe Systems Inc., San Jose, CA). Digital modifications of the images (brightness and contrast) were minimal so as not to affect the biological information. All double-labeled images were converted to magenta-green immunofluoresence to make this work more accessible to red-green color-blind readers.

Statistics
The significance of differences between the means in experimental groups and conditions was analyzed using parametric tests two-tailed unpaired Student's *t*-test or ordinary *one-way* ANOVA followed by *post hoc* Tukey multiple comparison test, using Prism (GraphPad Software Inc.). Significance levels indicated in all figures are as follows: *$P < 0.05$, **$P < 0.01$, ***$P < 0.001$, ****$P < 0.0001$. All data are presented as mean ± SEM (Standard error of mean). Finally, the *n* values reflect the final number of validated animals per group or the number of cells that were evaluated.

Supplementary References

Ampatzis, K., and Dermon, C.R. (2010). Regional distribution and cellular localization of beta2-adrenoceptors in the adult zebrafish brain (Danio rerio). J. Comp. Neurol. *518*, 1418–1441.
Ampatzis, K., Kentouri, M., and Dermon, C.R. (2008). Neuronal and glial localization of alpha(2A)-adrenoceptors in the adult zebrafish (Danio rerio) brain. J. Comp. Neurol. *508*, 72–93.
Angulo, M.C., Kozlov, A.S., Charpak, S., and Audinat, E. (2004). Glutamate released from glial cells synchronizes neuronal activity in the hippocampus. Journal of Neuroscience *24*, 6920–6927.
Barreiro-Iglesias, A., Mysiak, K.S., Adrio, F., Rodicio, M.C., Becker, C.G., Becker, T., and Anadón, R. (2013). Distribution of glycinergic neurons in the brain of glycine transporter-2 transgenic Tg(glyt2:Gfp) adult zebrafish: relationship to brain-spinal descending systems. J. Comp. Neurol. *521*, 389–425.
Berg, E.M., Bertuzzi, M., and Ampatzis, K. (2018). Complementary expression of calcium binding proteins delineates the functional organization of the locomotor network. Brain Struct. Funct. *223*, 2181–2196.
Bertuzzi, M., and Ampatzis, K. (2018). Spinal cholinergic interneurons differentially control motoneuron excitability and alter the locomotor network operational range. Sci Rep *8*, 1988.
Bertuzzi, M., Chang, W., and Ampatzis, K. (2018). Adult spinal motoneurons change their neurotransmitter phenotype to control locomotion. Proc. Natl. Acad. Sci. USA *115*, 9926-9933.
Böhm, U.L., Prendergast, A., Djenoune, L., Nunes Figueiredo, S., Gomez, J., Stokes, C., Kaiser, S., Suster, M., Kawakami, K., Charpentier, M., et al. (2016). CSF-contacting neurons regulate locomotion by relaying mechanical stimuli to spinal circuits. Nature Communications *7*, 10866.
Clemente, D., Porteros, Á., Weruaga, E., Alonso, J.R., Arenzana, F.J., Aijón, J., and Arévalo, R. (2004). Cholinergic elements in the zebrafish central nervous system: Histochemical and immunohistochemical analysis. J. Comp. Neurol. *474*, 75–107.

Djenoune, L., Desban, L., Gomez, J., Sternberg, J.R., Prendergast, A., Langui, D., Quan, F.B., Marnas, H., Auer, T.O., Rio, J.-P., et al. (2017). The dual developmental origin of spinal cerebrospinal fluid-contacting neurons gives rise to distinct functional subtypes. Sci Rep *7*, 719.

Hamilton, N.B., and Attwell, D. (2010). Do astrocytes really exocytose neurotransmitters? Nat. Rev. Neurosci. *11*, 227–238.

Higashijima, S.-I., Mandel, G., and Fetcho, J.R. (2004). Distribution of prospective glutamatergic, glycinergic, and GABAergic neurons in embryonic and larval zebrafish. J. Comp. Neurol. *480*, 1–18.

Kuscha, V., Barreiro-Iglesias, A., Becker, C.G., and Becker, T. (2012). Plasticity of tyrosine hydroxylase and serotonergic systems in the regenerating spinal cord of adult zebrafish. J. Comp. Neurol. *520*, 933–951.

McPherson, A.D., Barrios, J.P., Luks-Morgan, S.J., Manfredi, J.P., Bonkowsky, J.L., Douglass, A.D., and Dorsky, R.I. (2016). Motor Behavior Mediated by Continuously Generated Dopaminergic Neurons in the Zebrafish Hypothalamus Recovers after Cell Ablation. Current Biology *26*, 263–269.

Moly, P.K., Ikenaga, T., Kamihagi, C., Islam, A.F.M.T., and Hatta, K. (2014). Identification of initially appearing glycine-immunoreactive neurons in the embryonic zebrafish brain. Dev Neurobiol *74*, 616–632.

Montgomery, J.E., Wiggin, T.D., Rivera-Perez, L.M., Lillesaar, C., and Masino, M.A. (2016). Intraspinal serotonergic neurons consist of two, temporally distinct populations in developing zebrafish. Dev Neurobiol *76*, 673–687.

Mueller, T., Vernier, P., and Wullimann, M.F. (2006). A phylotypic stage in vertebrate brain development: GABA cell patterns in zebrafish compared with mouse. J Comp Neurol *494*, 620–634.

Ohnmacht, J., Yang, Y., Maurer, G.W., Barreiro-Iglesias, A., Tsarouchas, T.M., Wehner, D., Sieger, D., Becker, C.G., and Becker, T. (2016). Spinal motor neurons are regenerated after mechanical lesion and genetic ablation in larval zebrafish. Development *143*, 1464–1474.

Reimer, M.M., Sörensen, I., Kuscha, V., Frank, R.E., Liu, C., Becker, C.G., and Becker, T. (2008). Motor Neuron Regeneration in Adult Zebrafish. J. Neurosci. *28*, 8510–8516.

Rink, E., and Wullimann, M.F. (2004). Connections of the ventral telencephalon (subpallium) in the zebrafish (Danio rerio). Brain Res *1011*, 206–220.

Satou, C., Kimura, Y., Hirata, H., Suster, M.L., Kawakami, K., and Higashijima, S.-I. (2013). Transgenic tools to characterize neuronal properties of discrete populations of zebrafish neurons. Development *140*, 3927–3931.

Schneider, C.A., Rasband, W.S. and Eliceiri, K.W. (2012). NIH Image to ImageJ: 25 years of image analysis. Nature methods *9*, 671-675.
